# Draft Genome Sequence of *Staphylococcus succinus* subsp. *succinus* Type Strain DSM 14617, Isolated from Plant and Soil Inclusions within 25- to 35-Million-Year-Old Dominican Amber

**DOI:** 10.1128/genomeA.01521-16

**Published:** 2017-01-26

**Authors:** Hairui Zhou, Zhenyu Yao, Haihong Shi, Baixin Wang, Dong Li, Jie Hou, Shuxia Ma

**Affiliations:** Department of Medical School, Jiamusi University, Jiamusi, PR China

## Abstract

*Staphylococcus succinus* subsp. *succinus* type strain DSM 14617 was isolated from plant and soil inclusions within 25- to 35-million-year-old Dominican amber. The complete genome sequence of strain DSM 14617^T^ includes a genome of 2.88 Mb (32.94% G+C content) without any plasmids.

## GENOME ANNOUNCEMENT

*Staphylococcus succinus* was first described by L. H. Lambert in 1998 (1). Members of this group are widespread in nature. Many studies have reported the frequent isolation of *S. succinus* from various sources, such as cheese, dry or fermented meat products, the Dead Sea, and, occasionally, humans (2–6).

Type strain DSM 14617 was grown aerobically on Columbia blood agar base at 37°C for 24 h. Genomic DNA was extracted using the DNeasy blood and tissue kit (Qiagen, Germany). The quantity of DNA was measured by Cubit. Then, 10 μg of DNA was sent to Zhejiang University. One DNA library was generated (422-bp insert size, with the Illumina adapter at both ends) and then sequenced using an Illumina HiSeq 2000 platform, with a 2 × 100 paired-end sequencing strategy. A total of 16.58 Mb filtered reads were assembled into scaffolds using Velvet version 1.2.07 (7); a PAGIT flow (8) was then used to prolong the initial contigs and correct sequencing errors. Predicted genes were identified using Glimmer version 3.0 (9); tRNAscan-SE version 1.21 (10) was used to find tRNA genes, and ribosomal RNAs were found by using RNAmmer version 1.2 (11). HMMER version 3.0 (12) was used to annotate the predicted genes, and the KAAS server (13) was used to assign translated amino acids to the KEGG Orthology database, following the single-directional best-hit method. Translated genes were aligned with the COG database using NCBI BLASTp.

The draft genome sequence of strain DSM 14617^T^ revealed a genome size of 2.88 Mb and a G+C content of 32.94% (161 scaffolds with an *N*_50_ of 241,537 bp). These scaffolds contain 2,914 coding sequences and 96 RNAs. A total of 2,315 protein-coding genes were assigned as putative functional or hypothetical proteins, and 2,089 genes were categorized into COG functional groups.

### Accession number(s).

This whole-genome shotgun project has been deposited at DDBJ/EMBL/GenBank under the accession number LCSH00000000. The version described in this paper is the first version, LCSH01000000.
